# Application of Neuromodulation Techniques in the Treatment of Proprioceptive Disorders After Stroke: A Meta-Analysis of Randomized Controlled Trials

**DOI:** 10.1155/np/1705888

**Published:** 2025-09-16

**Authors:** Zhendong Zhang, Hu Yuming, Yu Huixian, Yumei Zhang

**Affiliations:** ^1^Beijing Tiantan Hospital, Capital Medical University, Beijing 100070, China; ^2^School of Nursing, Chinese Academy of Medical Sciences & Peking Union Medical College, No. 33 Badachu Road, Shi Jing Shan District, Beijing 100144, China

**Keywords:** neuromodulation, proprioception, RCT, rehabilitation, stroke

## Abstract

**Background and Objective:** After a stroke, proprioceptive disorders can impair patients' ability to perceive the speed and direction of movement accurately and promptly, as well as the spatial position of their limbs. This impairment often leads to motor dysfunction, including balance and postural control deficits, which severely affect patients' activities of daily living (ADLs) and quality of life. Neural plasticity is a key factor influencing poststroke functional recovery. In recent years, neuromodulation techniques targeting the enhancement of neural plasticity have emerged as a major research focus. This study aims to conduct a meta-analysis of the efficacy of neuromodulation techniques in treating proprioceptive disorders in stroke patients.

**Methods:** A systematic search was conducted in PubMed, Embase, the Cochrane Library, and Web of Science for studies investigating the effects of neuromodulation techniques on proprioceptive impairment in stroke patients. The search spanned from the inception of each database to December 2024. The primary outcome measure was the change in proprioception.

**Results:** In this meta-analysis, a total of nine randomized controlled trials (RCTs) were included. One study compared two different neuromodulation techniques, resulting in the extraction of 10 distinct datasets. Overall, 360 patients were involved in these studies. Specifically, 182 patients in the treatment group received neuromodulation techniques combined with conventional rehabilitation, while 178 patients in the control group received conventional rehabilitation alone. The meta-analysis revealed no significant difference in the efficacy of neuromodulation techniques combined with conventional rehabilitation compared to conventional rehabilitation alone(standardized mean difference [SMD] = 0.221,95% CI = −0.194 to 0.636, *p*=0.296). The subgroup analysis based on the stage of the stroke course revealed no significant differences between the two groups(acute stage: SMD = 0.303, 95% CI = −1.300 to 1.905, *p*=0.711; subacute stage: SMD = 0.351, 95% CI = −0.200 to 0.903, *p*=0.212; chronic phase: SMD = −0.047, 95% CI = −0.634 to 0.539, *p*=0.874). Subgroup analysis based on the types of neuromodulation techniques revealed that three specific techniques, electroacupuncture, repetitive transcranial magnetic stimulation (rTMS), and transcranial direct current stimulation (tDCS), were effective in improving proprioceptive disorders after stroke electroacupuncture group: SMD = −0.504, 95% CI = −1.006 to −0.002, *p*=0.049; rTMS group: SMD = 1.207, 95% CI = 0.246–2.168, *p*=0.014; tDCS group: SMD = 0.894, 95% CI = 0.323–1.465, *p*=0.002).

**Conclusion:** No statistically significant difference was found in the efficacy of neuromodulation techniques for treating proprioceptive disorders after stroke. Additionally, no significant differences were observed in the intervention across different stages of stroke. However, subgroup analysis indicated that electroacupuncture, rTMS, and tDCS may be effective in managing proprioceptive disorders poststroke. Therefore, it is recommended to prioritize these techniques in clinical practice.

## 1. Introduction

Proprioception refers to the perception of joint position, body movement, and spatial orientation [1]. This sensory feedback operates in real-time and is crucial for effective motor control [2]. For stroke patients, proprioception is pivotal for maintaining balance, coordination, motor control, and the ability to perform activities of daily living (ADLs) [3–6]. Alarmingly, up to 60% of stroke patients suffer from proprioceptive disorders [7]. Despite its importance, proprioception remains underemphasized in both clinical practice and the majority of stroke rehabilitation studies [8, 9], unlike motor function, there is a lack of consensus regarding standardized evaluation methods and targeted rehabilitation approaches for proprioception.

The International Neuromodulation Society defines neuromodulation as “altering neural activity by specifically delivering stimuli (such as electrical stimulation or chemical agents) to specific parts of the nervous system within the body” [10]. Given that poststroke functional recovery is influenced by neural plasticity, neuromodulation techniques targeting the enhancement of neural plasticity have emerged as a major research focus in recent years. These techniques are categorized into invasive and noninvasive methods. Invasive neuromodulation techniques, such as vagus nerve stimulation (VNS), deep brain stimulation (DBS), spinal cord stimulation (SCS), motor cortex stimulation (MCS), and peripheral nerve stimulation (PNS), have demonstrated significant potential in promoting stroke rehabilitation. Noninvasive neuromodulation techniques, including transcutaneous VNS (tVNS), transcranial magnetic stimulation (TMS), and transcranial direct current stimulation (tDCS), have been shown to improve motor function in stroke patients [11].

However, the efficacy of neuromodulation techniques in treating proprioceptive disorders after stroke remains inconclusive. Results from relevant clinical studies are inconsistent, and the optimal timing for intervention remains unclear. Therefore, this study aims to conduct a meta-analysis to evaluate the efficacy of neuromodulation techniques for proprioceptive disorders poststroke.

## 2. Methods

This meta-analysis was presented according to the Preferred Reporting Items for Systematic Reviews and Meta-Analyses (PRISMA) guidelines and registered in the PROSPERO database (Registration Number CRD420251017532, https://www.crd.york.ac.uk/prospero/) [12].

### 2.1. Search Strategy

Published studies were identified from PubMed, Embase, the Cochrane Library, and Web of Science using search terms related to “stroke,” “proprioception,” “neuromodulation,” “rehabilitation,” “RCT,” and their synonyms, from the inception of each database up to December 2024. The full search strategy is detailed in the Appendix A. No restrictions were applied regarding language or publication date. Additionally, references from identified studies and gray literature were manually searched to ensure comprehensiveness.

### 2.2. Inclusion and Exclusion Criteria

Inclusion criteria are as follows:

1. Stroke patients aged 18 years or older.
2. The primary intervention utilized was neuromodulation technology.
3. Outcome measures, including tests of position perception and movement perception, the Nottingham Sensory Assessment Scale, and the sensory component of the Fugl–Meyer Assessment.
4. Randomized controlled trials (RCTs).

Exclusion criteria are as follows:

1. Animal studies and Pharmacological trial.
2. Retrospective studies and cohort studies.
3. Duplicate publications.
4. Meta-analysis, review, meeting extracts, case reports, and letters.
5. The data are incomplete or the full text is unavailable, and attempts to contact the author have been unsuccessful.

### 2.3. Screening and Data Extraction

Articles were imported into EndNote X21 and screened by two independent researchers according to the eligibility criteria. Any disagreements were resolved through discussion with a third researcher. The extracted data included general study characteristics (author, year of publication, country, and study type) and patient characteristics (age, number of patients in each group, and type of disease).

### 2.4. Quality Assessment

The risk of bias for each outcome of the included RCTs was assessed using Version 2 of the Cochrane risk-of-bias tool for randomized trials (RoB 2). The specific domains evaluated included the randomization process, deviations from intended interventions, missing outcome data, outcome measurement, and selection of reported results [13]. Two authors independently conducted the quality assessment, with discrepancies resolved by a third author.

### 2.5. Statistical Analysis

The meta-analysis was performed using Stata 18 MP. Given that proprioception is a continuous variable and different assessment criteria were used, the standardized mean difference (SMD) was employed for data synthesis. Point estimates and 95% confidence intervals were calculated for each effect size. Additionally, the *I*^2^ statistic was used to assess heterogeneity among the studies.

The heterogeneity of the included studies was assessed using the *χ*^2^ test (*α* = 0.1), and the degree of heterogeneity was quantitatively determined by the *I*^2^ statistic simultaneously: *I*^2^ < 50% was considered mild heterogeneity, *I*^2^ = 50%–< 75% was considered moderate heterogeneity, and *I*^2^ ≥ 75% was considered high heterogeneity [14]. When mild heterogeneity was present, a fixed-effect model was used for meta-analysis. When moderate heterogeneity was detected, potential sources of heterogeneity were explored. After excluding the influence of significant clinical heterogeneity, a random-effects model was employed for meta-analysis. When high heterogeneity was observed, a general statistical description of the study results was provided [15].

Subgroup analyses were conducted to explore the potential sources of heterogeneity. Sensitivity analyses were performed to evaluate the robustness of the test results. Additionally, Egger's test was utilized to assess publication bias.

## 3. Results

### 3.1. Article Search and Screening

**Figure 1:**
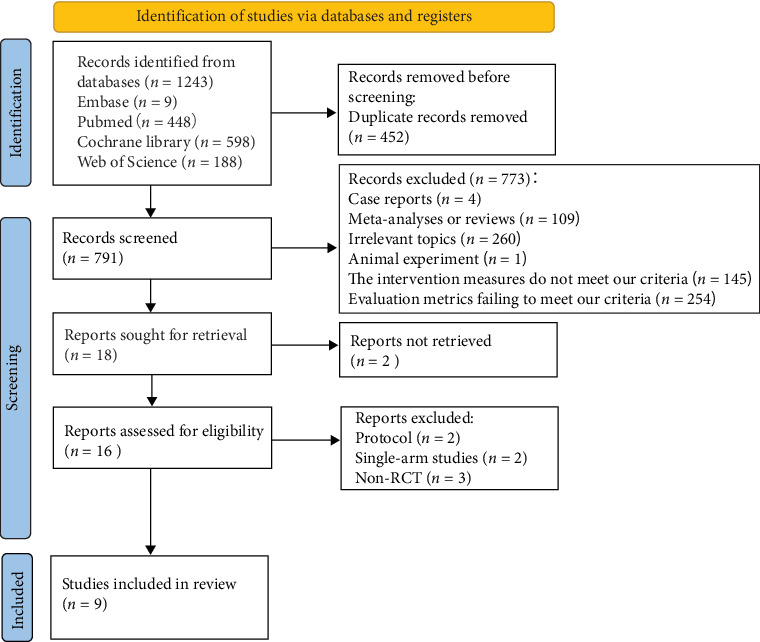
PRISMA 2020 flow diagram.

In accordance with the search strategy, a total of 1243 articles were initially identified. After removing 452 duplicate records, the titles, abstracts, and full texts of the remaining 791 articles were reviewed. Based on the predefined inclusion and exclusion criteria, nine articles were ultimately selected for the meta-analysis. This review adheres to the PRISMA statement [12] (Figure 1).

To establish a robust evidence base, we included nine RCTs addressing different aspects of neuromodulation techniques for proprioceptive disorders after stroke as follows:

Study 1 [16] implemented a double-blind, allocation-concealed trial design comparing transcutaneous electrical nerve stimulation (TENS) and repetitive TMS (rTMS), verifying synergistic effects of neuromodulation techniques.

Study 2 [17] was the only investigation employing a paired-sample randomized crossover trial to verify TENS efficacy for proprioceptive impairment in chronic stroke patients.

Study 3 [18] constituted the sole RCT using electrical stimulation therapy, introducing an additional subgroup for our subgroup analysis.

Study 4 [19] exclusively enrolled patients with unilateral middle cerebral artery infarction.

Study 5 [20] included acute-stage stroke patients, enhancing the subgroup analysis of stroke course stages.

Study 6 [21] was identified through our literature search as the first investigation examining tDCS efficacy for stroke-related proprioceptive impairment.

Study 7 [22] verified synergistic effects of TENS versus traditional Bobath rehabilitation techniques for stroke proprioceptive disorders.

Study 8 [23] confirmed synergistic effects of TENS compared with conventional rehabilitation training (e.g., treadmill training) for stroke proprioceptive impairment.

Study 9 [24] represented the first RCT of electroacupuncture for stroke proprioception disorders, providing proprioception score data at 3-month post-treatment follow-up.

### 3.2. General Characteristics of Included Studies

The nine included studies were all RCTs, with one study from China, one from Turkey, three from South Korea, one from Germany, one from the United Kingdom, one from Brazil, and one from Belgium (Table 1).

The studies included one on electroacupuncture [24], four on TENS [16, 17, 22, 23], one on repetitive sensory stimulation [19], one on electrical stimulation therapy [18], one on rTMS [16], and two on tDCS [20, 21]. Freitas Zanona et al. [16] concurrently compared the efficacy of rTMS and TENS on proprioceptive disorders in stroke patients within a single study. Regarding the stages of stroke, two studies focused on patients in the acute phase [20, 24], four studies targeted the subacute phase [16, 19, 21, 23], and three studies examined the chronic phase [17, 18, 22]. A total of 360 patients were included in the meta-analysis, comprising 182 patients in the treatment group who received neuromodulation techniques combined with conventional rehabilitation, and 178 patients in the control group who received conventional rehabilitation alone.

### 3.3. Quality Assessment of Included Studies

**Figure 2:**
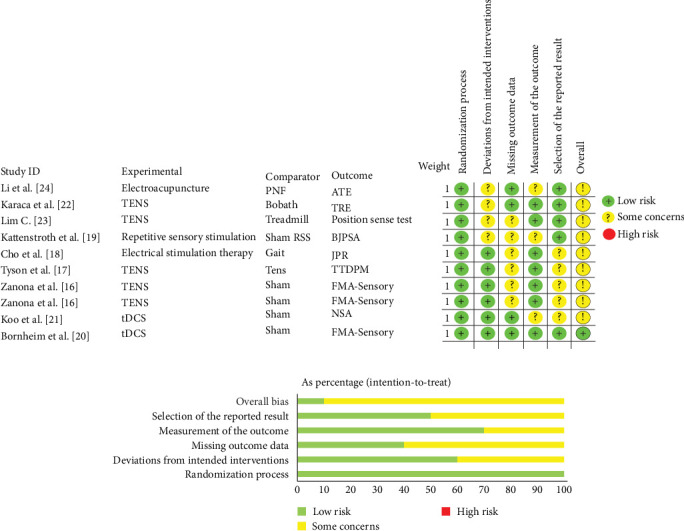
Summary of risk of bias for included randomized controlled trials (RoB 2): single study dimension analysis (a) and overall domain distribution (b).

The methodological quality of all included RCTs was rigorously assessed using the Cochrane Risk of Bias 2 (RoB 2) tool. Among the nine studies, one was rated as having a low risk of bias, with appropriate randomization and blinding procedures. The remaining eight studies exhibited some risks of uncertainty, primarily due to insufficient information regarding the integrity of outcome data, measurement bias, and reporting bias. Overall, the quality of the included studies was deemed moderate. However, more stringent blinding and allocation concealment designs may be warranted to enhance study rigor. The risk of bias assessment for the included studies is presented in Figure 2.

### 3.4. Meta-Analysis

**Figure 3:**
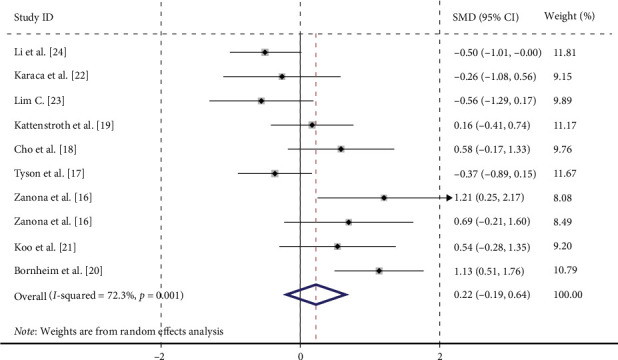
Forest plot of the therapeutic effect of neuromodulation techniques on proprioceptive disorders poststroke.

Given the varying criteria for assessing proprioception across studies, Hedges' *g* was employed to calculate the SMD for meta-analysis. Effect sizes were pooled from 10 datasets (*n* = 360) derived from nine studies (Figure 3). The heterogeneity test revealed significant heterogeneity (*p*=0.001, *I* = 72.3%), prompting the use of a random-effects model. The meta-analysis indicated no statistically significant difference in the efficacy of neuromodulation techniques for treating proprioceptive impairment after stroke (SMD = 0.221, 95% CI = −0.194 to 0.636, *p*=0.296).

#### 3.4.1. Sensitivity Analysis

**Figure 4:**
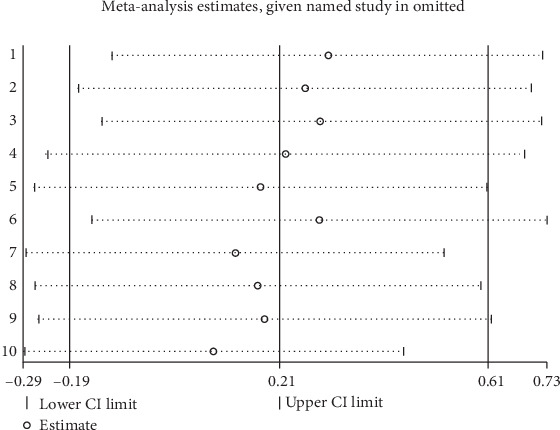
Sensitivity analysis chart.

Regardless of which study was excluded, the pooled results of the remaining studies consistently showed no significant difference and remained statistically nonsignificant. This demonstrates the robustness of the meta-analysis results. (The results of the sensitivity analysis are presented in Figure 4).

#### 3.4.2. Subgroup Analysis

**Figure 5:**
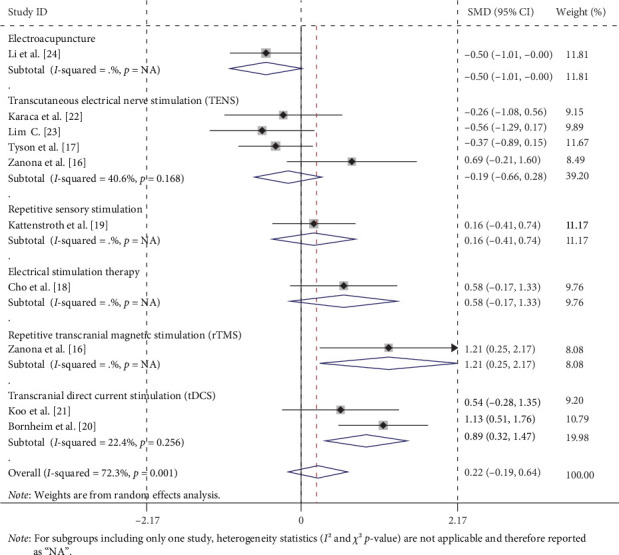
Forest plots stratified by neuromodulation technique types.

Subgroup analyses were conducted based on the type of neuromodulation technique (Figure 5) as follows:

Electroacupuncture: One study [24] reported an SMD of −0.504 (95% CI = −1.006 to −0.002, *p*=0.049), indicating that electroacupuncture was significantly more effective than the control. However, this subgroup result, based solely on a single study, should be considered exploratory and interpreted cautiously due to limited reliability.

TENS: Four studies [16, 17, 22, 23] yielded a combined SMD of −0.194 (95% CI = −0.664 to 0.275, *p*=0.417), showing no significant difference between the TENS intervention and the control group. Heterogeneity was mild (*I*^2^ = 40.6%, *p*=0.168), but not statistically significant.

Repetitive sensory stimulation: One study [19] showed an SMD of 0.164 (95% CI = −0.415 to 0.743, *p*=0.579), with no significant effect. In addition, this subgroup result is based on only a single study and should be considered exploratory and interpreted with caution due to limited reliability.

Electrical stimulation therapy: One study [18] reported an SMD of 0.581 (95% CI = −0.166 to 1.327, *p*=0.127), which was not statistically significant. However, this subgroup result, based solely on a single study, should be considered exploratory and interpreted cautiously due to limited reliability.

rTMS: One study [16]reported an SMD of 1.207, (95% CI = 0.246 to 2.168, *p*=0.014), indicating that rTMS was significantly more effective than the control. This subgroup result is also based on only a single study and is also an exploratory analysis due to limited reliability.

tDCS: Two studies [20, 21] had a combined SMD of 0.894 (95% CI = 0.323 to 1.465, *p*=0.002), showing that tDCS was significantly more effective than the control. Heterogeneity was mild (*I*^2^ = 22.4%, *p*=0.256), but not statistically significant.

**Figure 6:**
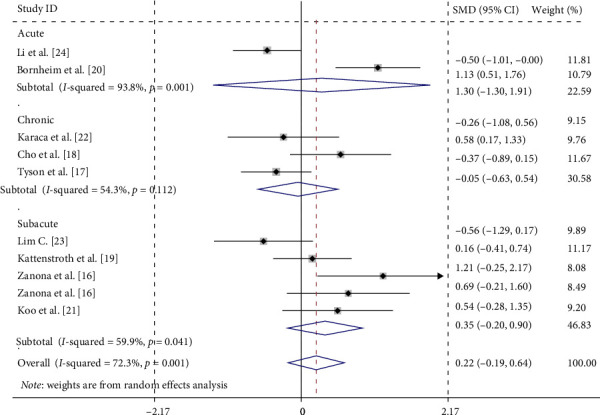
Forest plots of subgroup analyses by stroke stage.

Subgroup analyses were conducted based on the stage of the stroke course (Figure 6) as follows:

Acute stage: Two studies [20, 24] reported an SMD of 0.303 (95% CI = −1.300 to 1.905, *p*=0.711), indicating no significant effect of neuromodulation intervention in the acute stage. High heterogeneity was observed among these studies (*I*^2^ = 93.8%, *p*=0.001).

Subacute stage: Four studies [16, 19, 21, 23] yielded a combined SMD of 0.351 (95% CI = −0.200 to 0.903, *p*=0.212), showing no significant effect of neuromodulation intervention in the subacute stage. Moderate heterogeneity was noted (*I*^2^ = 59.9%, *p*=0.041).

Chronic phase: Three studies [17, 18, 22] reported an SMD of −0.047 (95% CI = −0.634 to 0.539, *p*=0.874), suggesting no significant effect of neuromodulation intervention in the chronic phase. Moderate heterogeneity was observed (*I*^2^ = 54.3%, *p*=0.112).

## 4. Discussion

Stroke is characterized by high rates of disability, recurrence, and mortality, and it remains the leading cause of adult disability [25]. Reports indicate that up to 60% of stroke patients experience proprioceptive disorders [7], which are often associated with significant declines in motor function. In a meta-analysis, Yu et al. [3] identified a correlation between proprioceptive and motor dysfunction in stroke patients. Proprioceptive disorders can impair stroke patients' ability to perceive movement speed and direction promptly and accurately, preventing swift adjustments during functional activities. This leads to balance dysfunction and postural adjustment disorders, ultimately reducing stroke patients' capacity for ADLs and diminishing their quality of life [26].

Our meta-analysis is pioneering in exploring whether neuromodulation technology can enhance conventional rehabilitation treatments for stroke patients with proprioceptive disorders. This innovative approach offers valuable insights for clinical practice.

**Figure 7:**
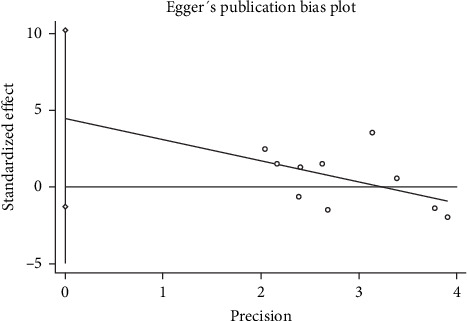
Funnel plot for publication bias assessment of included studies.

Our meta-analysis of nine RCTs revealed that neuromodulation techniques had no statistically significant effect on the recovery of proprioceptive impairment in stroke patients (SMD = 0.221, 95% CI = −0.194 to 0.636, *p*=0.296). Potential reasons for this finding include the following:

1. Limited sample size: Only nine studies were included in the meta-analysis, with a total sample size of 360 participants. The combined results may lack sufficient statistical power to detect a true effect.
2. Publication bias: Although Egger's test did not identify significant publication bias (*p*=0.112), the funnel plot (Figure 7) exhibited obvious asymmetry, with the distribution of study points deviating from an ideal symmetrical funnel shape. Further verification using Begg's test revealed statistically significant publication bias (*p*=0.040 before correction, *p*=0.049 after correction). This suggests that the meta-analysis may be at risk of publication bias, with some studies having small or negative effect sizes potentially missing from the included studies, possibly due to nonpublication.
3. Significant heterogeneity: High heterogeneity was observed among the included studies (*I*^Two^ = 72.3%), which may limit the accuracy of the pooled estimates.

The meta-analysis revealed substantial heterogeneity across the nine included studies (*I*^2^ = 72.3%), potentially affecting the robustness of the results. Given the limited number of eligible studies, further reduction of heterogeneity was not feasible. To mitigate this limitation, we conducted sensitivity and subgroup analyses stratified by stroke course stage and neuromodulation technique, aiming to identify heterogeneity sources and evaluate their influence on the pooled estimates.

We examined the potential sources of heterogeneity among the included studies are as follows:

1. Treatment cycle: Following a stroke, patients typically undergo significant reorganization of brain function, which involves the reconnection of neural pathways and the formation of new neural networks. This reorganization is crucial for the recovery of various functions [27]. While repetition is key to enhancing synaptic efficacy [28], a longer duration of rehabilitation intervention may yield better outcomes [29].
2. Differences in technology types: Various neuromodulation techniques operate through distinct mechanisms. Peripheral stimulation methods, such as repetitive sensory stimulation, TENS, and electrical stimulation therapy, promote nerve function remodeling by stimulating the peripheral nervous system [30]. In contrast, central regulation techniques like rTMS and tDCS improve poststroke dysfunction by precisely targeting and applying specific stimulation to damaged brain areas or functional brain regions. These techniques increase cortical excitability in the affected hemisphere and decrease it in the healthy hemisphere, thereby rebalancing cortical excitability and reducing interhemispheric inhibition [31–33].
3. Inconsistent direction of effect size: The forest plot combining all effect sizes (Figure 3) revealed that some studies showed the efficacy of combining neuromodulation techniques with conventional rehabilitation, while others indicated that conventional rehabilitation alone was more effective.
4. Methodological limitations: Among the included studies, one [24] did not mention blinding, and three [21, 22, 24] did not mention allocation concealment, introducing methodological limitations.
5. Insensitivity of outcome indicators: Compared to other included studies, the subjective scales (such as FMA-Sensory, NSA) used in three studies [16, 20, 21] may not be sensitive enough to capture subtle changes in proprioception.

In our meta-analysis, despite the small and nonsignificant effect size, the point estimates indicated a beneficial trend toward improved proprioception (SMD = 0.221 > 0), which was corroborated by some positive findings in subgroup analyses. Specifically, the subgroup analysis based on the stage of the stroke course (Figure 6) revealed no significant differences in the efficacy of neuromodulation techniques compared to conventional rehabilitation techniques across the acute, subacute, and chronic stages. This lack of significance may be attributed to the limited sample size. However, the subgroup analysis of specific neuromodulation techniques, including electroacupuncture, rTMS, and tDCS (Figure 5), demonstrated significant improvements in proprioceptive disorders among stroke patients.

In our meta-analysis, only one study examined the Electroacupuncture technique. The subgroup analysis revealed an SMD of −0.504 (95% CI = −1.006 to −0.002, *p*=0.049), indicating that Electroacupuncture was significantly more effective than the control group. Given the small sample size, these findings are preliminary and warrant verification in larger studies. Electroacupuncture has been shown to promote neurogenesis [34], and enhance cerebral blood supply by acting on vasoactive mediators and stimulating cerebral angiogenesis [35, 36], suggesting its potential benefits in improving proprioception in stroke patients.

rTMS and tDCS are noninvasive neuromodulation techniques that can selectively and specifically influence brain plasticity and guide brain network reorganization after stroke [31–33]. The transcallosal disinhibition hypothesis [37] posits that following a stroke, the cortical excitability of the infarcted hemisphere is reduced, diminishing its normal inhibitory effect on the unaffected hemisphere and/or causing the unaffected hemisphere to exert excessive influence on the affected hemisphere. rTMS modulates cortical excitability in specific brain regions through distinct frequencies: high-frequency rTMS (frequency > 1 Hz) enhances cortical excitability, while low-frequency rTMS (frequency ≤ 1 Hz) diminishes it [38]. Freitas Zanona et al. [39]. applied 10 Hz rTMS to the affected primary somatosensory cortex (S1) area in stroke patients, revealing improved primary somatosensory cortex (S1) excitability and reduced interhemispheric asymmetry, thereby enhancing sensorimotor function. In animal studies, rTMS has been shown to affect neurotransmitter or receptor levels, alter neuronal electrophysiological properties, and reregulate the expression of excitatory and inhibitory neurotransmitters, thereby modulating long-term potentiation or depression and enhancing synaptic plasticity [40, 41].

In this meta-analysis, only one RCT was included for rTMS (SMD = 1.207, 95% CI = 0.246 to 2.168, *p*=0.014), which demonstrated significant efficacy compared to the control group. Despite this promising result, multicenter RCTs are recommended to further verify the long-term effects of rTMS on proprioceptive impairment poststroke.

The tDCS is a noninvasive technique that modulates cortical excitability through weak direct current stimulation. We included two clinical studies on tDCS neuromodulation technology. The pooled SMD from the subgroup analysis was 0.894 (95% CI = 0.323 to 1.465, *p*=0.002), indicating that tDCS intervention was significantly more effective than conventional rehabilitation treatment. Heterogeneity was mild (*I*^2^ = 22.4%, *p*=0.256) and did not reach statistical significance. Possible mechanisms include the following:

1. Functional connectivity: tDCS effects extend beyond the stimulated area to involve nearby brain regions, altering functional connections between different brain areas. This promotes functional integration across various cortical regions and enhances the functional connectivity of motor centers and neural networks [42].
2. Neuronal modulation: tDCS delivers continuous weak currents to the scalp via positive or negative electrodes, affecting the resting membrane potential of neurons. It also modulates synaptic functions of various receptors, synapses, and neurotransmitters, thereby enhancing brain plasticity [43].Studies have shown that tDCS changes the firing threshold of cortical neurons [44], increases neuronal excitability, and improves cortical and somatic sensation in stroke patients [21]. This enhances cortical excitability and connectivity, improving neuronal function and structural plasticity [45], and thereby enhances patients' ability to regulate and process sensory and motor functions [46].
3. Cortical rebalancing: tDCS can rebalance cortical excitability and improve interhemispheric inhibition [32], anodal tDCS (positive electrode) enhances cortical excitability in the target brain area, while cathodal tDCS (negative electrode) has the opposite effect [31].

Given these mechanisms, tDCS appears to be a promising neuromodulation technique. We recommend conducting more high-quality RCTs with larger sample sizes to verify both the short-term and long-term efficacy of tDCS.

While lesion location, medical history, and rehabilitation intensity are established determinants of stroke recovery outcomes, our meta-analysis was limited by both the paucity of included studies and insufficient reporting of these confounding variables in the existing literature. Future investigations on proprioceptive rehabilitation in stroke should prioritize comprehensive documentation of these factors to enable more robust meta-analyses examining their influence on proprioceptive impairment.

## 5. Limitations

This study has several limitations that should be acknowledged. First, the meta-analysis may be susceptible to publication bias, which could stem from either the limited number of eligible studies or the potential omission of unpublished negative results. Second, in some subgroup analyses, the findings were disproportionately influenced by a single study due to the scarcity of available original research. This underscores the need for additional high-quality studies to validate these subgroup results. Third, substantial heterogeneity was observed across studies. While we conducted thorough analyses to explore potential sources of heterogeneity, the fundamental constraint posed by the limited number of clinical investigations in this field remains an important consideration. Fourth, certain assessment tools employed in the included studies (e.g., FMA-Sensory, NSA) may lack the sensitivity required to detect subtle proprioceptive impairments. Given that these scales were integral to the original study designs, alternative measures could not be retrospectively applied. Future research should prioritize the development and implementation of more sensitive proprioceptive assessment methods.

## 6. Conclusion

Our meta-analysis demonstrated no statistically significant difference in overall efficacy among various neuromodulation techniques for poststroke proprioceptive impairment. Subgroup analyses revealed two key findings: (1) intervention timing (the stroke stage) did not significantly affect outcomes, though this conclusion requires cautious interpretation due to limited sample sizes and potential publication bias and (2) peripheral electroacupuncture, central rTMS, and tDCS emerged as potentially effective approaches, which clinicians may consider prioritizing in rehabilitation protocols.

Future studies should prioritize conducting high-quality RCTs with rigorous methodological design to validate the therapeutic efficacy of electroacupuncture, rTMS, and tDCS for poststroke proprioceptive disorders. These investigations should incorporate both conventional clinical assessments and more sensitive objective evaluation methods, including comprehensive neuroelectrophysiological testing (e.g., somatosensory evoked potentials) and functionally relevant task-specific performance measures. Such an approach would enable systematic exploration of how different stimulation parameters (intensity, frequency, duration), treatment protocols (session frequency, total intervention period), and combined neuromodulation strategies influence therapeutic outcomes. Additionally, advanced neuroimaging techniques, particularly functional MRI (fMRI), should be employed to investigate the neural mechanisms underlying treatment-induced neuroplastic changes and functional recovery.

## Figures and Tables

**Table 1:** General characteristics of included articles.

First author	Year	Design	Country	Treat/control (*N*)	Treat/control (age)	Types of stroke	Type of techniques	Phase	Outcome measures
Li et al. [24]	2019	RCT	China	31/32	56 ± 8/57 ± 10	Hemorrhagic and ischemic strokes	Electroacupuncture	Acute	ATE
Karaca and Kılınç [22]	2024	RCT	Turkey	11/12	30–78/46–80	Hemorrhagic and ischemic strokes	TENS	Chronic	TRE
Lim [23]	2019	RCT	Korea	15/15	62 ± 7.3/59.61 ± 6.77	Hemorrhagic and ischemic strokes	TENS	Subacute	Position sense test
Kattenstroth et al. [19]	2018	RCT	Germany	23/23	34–86/43–89	Ischemic stroke	RSS	Subacute	BJPSA
Cho et al. [18]	2022	RCT	Korea	18/12	51.8 ± 12/55 ± 10.9	Not specified	Electrical stimulation therapy	Chronic	JPR
Tyson et al. [17]	2013	RCT	UK	29/29	64.5 ± 12.6/64.5 ± 12.6	Not specified	TENS	Chronic	TTDPM
Freitas Zanona et al. [16]	2022	RCT	Brazil	10/10	62.6 ± 7.9/61.6 ± 11.3	Hemorrhagic and ischemic strokes	rTMS	Subacute	FMA-Sensory
Freitas Zanona et al. [16]	2022	RCT	Brazil	10/10	62.1 ± 11.4/61.6 ± 11.3	Hemorrhagic and ischemic strokes	TENS	Subacute	FMA-Sensory
Koo et al. [21]	2018	RCT	Korea	12/12	52.42 ± 3.23/58.67 ± 3.4	Hemorrhagic and ischemic strokes	tDCS	Subacute	NSA
Bornheim et al. [20]	2020	RCT	Belgium	23/23	62.48 ± 11.86/63.48 ± 12.94	Ischemic stroke	tDCS	Acute	FMA-Sensory

*Note*: FMA-Sensory, Fugl–Meyer assessment for sensory function; JPR, joint position reproduction test.

Abbreviations: ATE, average trace error; BJPSA, bochum joint position sense assessment; NSA, Nottingham Sensory Assessment; RSS, repetitive sensory stimulation; rTMS, repetitive transcranial magnetic stimulation; tDCS, transcranial direct current stimulation; TENS, transcutaneous electrical nerve stimulation; TRE, trunk reposition error; TTDPM, threshold to detection of passive motion.

## Data Availability

The data that support the findings of this study are available from the corresponding author upon reasonable request.

## Appendix A: The Cochrane Library Search Strategy and Literature Quantity Documentation

1. MeSH descriptor: [Stroke] explode all trees——17639.
2. (Strokes):ti,ab,kw OR (Cerebrovascular Accident):ti,ab,kw OR (Cerebrovascular Accidents):ti,ab,kw OR (Cerebral Stroke):ti,ab,kw OR (Cerebral Strokes):ti,ab,kw——78321.
3. (Stroke, Cerebral):ti,ab,kw OR (Strokes, Cerebral):ti,ab,kw OR (Cerebrovascular Apoplexy):ti,ab,kw OR (Apoplexy, Cerebrovascular):ti,ab,kw OR (Vascular Accident, Brain):ti,ab,kw——8944.
4. (Brain Vascular Accident):ti,ab,kw OR (Brain Vascular Accidents):ti,ab,kw OR (Vascular Accidents, Brain):ti,ab,kw OR (Cerebrovascular Stroke):ti,ab,kw OR (Cerebrovascular Strokes):ti,ab,kw——19224.
5. (Stroke, Cerebrovascular):ti,ab,kw OR (Strokes, Cerebrovascular):ti,ab,kw OR (Apoplexy):ti,ab,kw OR (CVA [Cerebrovascular Accident]):ti,ab,kw OR (CVA [Cerebrovascular Accident]):ti,ab,kw——19619.
6. (Stroke, Acute):ti,ab,kw OR (Acute Stroke):ti,ab,kw OR (Acute Strokes):ti,ab,kw OR (Strokes, Acute):ti,ab,kw OR (Cerebrovascular Accident, Acute):ti,ab,kw——20852.
7. (Acute cErebrovascular Accident):ti,ab,kw OR (Acute Cerebrovascular Accidents):ti,ab,kw OR (Cerebrovascular Accidents, Acute):ti,ab,kw——4905.
8. #1 or #2 or #3 or #4 or #5 or #6 or #7——79098.
9. MeSH descriptor: [Brain Infarction] explode all trees——1896.
10. (Brain Infarctions):ti,ab,kw OR (Infarction, Brain):ti,ab,kw OR (Infarctions, Brain):ti,ab,kw OR (Brain Infarct):ti,ab,kw OR (Brain Infarcts):ti,ab,kw——5988.
11. (Infarct, Brain):ti,ab,kw OR (Infarcts, Brain):ti,ab,kw OR (Venous Infarction, Brain):ti,ab,kw OR (Brain Venous Infarction):ti,ab,kw OR (Brain Venous Infarctions):ti,ab,kw——5988.
12. (Infarction, Brain Venous):ti,ab,kw OR (Infarctions, Brain Venous):ti,ab,kw OR (Venous Infarctions, Brain):ti,ab,kw OR (Brain Infarction, Venous):ti,ab,kw OR (Brain Infarctions, Venous):ti,ab,kw——196.
13. (Infarctions, Venous Brain):ti,ab,kw OR (Infarction, Venous Brain):ti,ab,kw OR (Venous Brain Infarction):ti,ab,kw OR (Venous Brain Infarctions):ti,ab,kw OR (Anterior Cerebral Circulation Infarction):ti,ab,kw——499.
14. (Infarction, Anterior Cerebral Circulation):ti,ab,kw OR (Anterior Circulation, Brain Infarction):ti,ab,kw OR (Anterior Circulation Infarction, Brain):ti,ab,kw OR (Brain Infarction, Anterior Circulation):ti,ab,kw OR (Infarction, Anterior Circulation, Brain):ti,ab,kw——398.
15. (Infarction, Brain, Anterior Circulation):ti,ab,kw OR (Brain Infarction, Posterior Circulation):ti,ab,kw OR (Posterior Circulation Brain Infarction):ti,ab,kw OR (Posterior Circulation Infarction, Brain):ti,ab,kw OR (Infarction, Brain, Posterior Circulation):ti,ab,kw——321.
16. (Infarction, Posterior Circulation, Brain):ti,ab,kw——81.
17. #9 or #10 or #11 or #12 or #13 or #14 or #15 or #16——7025.
18. MeSH descriptor: [Cerebral Hemorrhage] explode all trees——1590.
19. (Hemorrhage, Cerebral):ti,ab,kw OR (Cerebral Hemorrhages):ti,ab,kw OR (Hemorrhages, Cerebral):ti,ab,kw OR (Intracerebral Hemorrhage):ti,ab,kw OR (Hemorrhage, Intracerebral):ti,ab,kw——7725.
20. (Hemorrhages, Intracerebral):ti,ab,kw OR (Intracerebral Hemorrhages):ti,ab,kw OR (Hemorrhage, Cerebrum):ti,ab,kw OR (Cerebrum Hemorrhage):ti,ab,kw OR (Cerebrum Hemorrhages):ti,ab,kw——3141.
21. (Hemorrhages, Cerebrum):ti,ab,kw OR (Brain Hemorrhage, Cerebral):ti,ab,kw OR (Brain Hemorrhages, Cerebral):ti,ab,kw OR (Cerebral Brain Hemorrhage):ti,ab,kw OR (Cerebral Brain Hemorrhages):ti,ab,kw——3392.
22. (Hemorrhage, Cerebral brain):ti,ab,kw OR (Hemorrhages, Cerebral Brain):ti,ab,kw OR (Cerebral Parenchymal Hemorrhage):ti,ab,kw OR (Cerebral Parenchymal Hemorrhages):ti,ab,kw OR (Hemorrhage, Cerebral Parenchymal):ti,ab,kw——3430.
23. (Hemorrhages, Cerebral Parenchymal):ti,ab,kw OR (Parenchymal Hemorrhage, Cerebral):ti,ab,kw OR (Parenchymal Hemorrhages, Cerebral):ti,ab,kw——126.
24. #18 or #19 or #20 or #21 or #22 or #23——7735.
25. #8 or #17 or #24——84226.
26. MeSH descriptor: [Proprioception] explode all trees——5091.
27. (Position Sense):ti,ab,kw OR (Sense, Position):ti,ab,kw OR (Posture Sense):ti,ab,kw OR (Sense, Posture):ti,ab,kw OR (Sense Of Position):ti,ab,kw——2913.
28. (Vestibular Sense):ti,ab,kw OR (Sense, Vestibular):ti,ab,kw OR (Labyrinthine Sense):ti,ab,kw OR (Sense, Labyrinthine):ti,ab,kw OR (Sense of Equilibrium):ti,ab,kw——135.
29. (Equilibrium Sense):ti,ab,kw——48.
30. MeSH descriptor: [Kinesthesis] explode all trees——180.
31. (Kinestheses):ti,ab,kw OR (Kinesthetic Sense):ti,ab,kw OR (Kinesthetic Senses):ti,ab,kw OR (Kinesthesia):ti,ab,kw OR (Kinesthesias):ti,ab,kw——150.
32. (Movement Sensation):ti,ab,kw OR (Movement Sensations):ti,ab,kw——955.
33. #26 or #27 or #28 or #29 or #30 or #31 or #32——8734.
34. MeSH descriptor: [Exercise Therapy] explode all trees——22095.
35. (Rehabilitation Exercise):ti,ab,kw OR (Exercise, Rehabilitation):ti,ab,kw OR (Exercises, Rehabilitation):ti,ab,kw OR (Rehabilitation Exercises):ti,ab,kw OR (Therapy, Exercise):ti,ab,kw——69567.
36. (Exercise Therapies):ti,ab,kw OR (Therapies, Exercise):ti,ab,kw OR (Remedial Exercise):ti,ab,kw OR (Exercise, Remedial):ti,ab,kw OR (Exercises, Remedial):ti,ab,kw——2469.
37. (Remedial Exercises):ti,ab,kw AND (Habilitation):ti,ab,kw——0.
38. MeSH descriptor: [Rehabilitation] explode all trees——55262.
39. #34 or #35 or #36 or #37 or #38——104159.
40. #25 and #33 and #39——598.
